# Playful brains: a possible neurobiological pathway to cognitive health in aging

**DOI:** 10.3389/fnhum.2025.1490864

**Published:** 2025-02-07

**Authors:** Yulia Golland, Boaz M. Ben-David, Mara Mather, Shoshi Keisari

**Affiliations:** ^1^Dina Recanati School of Medicine, Reichman University, Herzliya, Israel; ^2^Baruch Ivcher School of Psychology, Reichman University, Herzliya, Israel; ^3^Department of Speech-Language Pathology, University of Toronto, Toronto, ON, Canada; ^4^KITE, Toronto Rehabilitation Institute, University Health Networks, Toronto, ON, Canada; ^5^Leonard Davis School of Gerontology, University of Southern California, Los Angeles, CA, United States; ^6^Department of Psychology, University of Southern California, Los Angeles, CA, United States; ^7^Department of Biomedical Engineering, University of Southern California, Los Angeles, CA, United States; ^8^School of Creative Arts Therapies, Faculty of Social Welfare and Health Sciences, University of Haifa, Haifa, Israel; ^9^The Center for Research and Study of Aging, University of Haifa, Haifa, Israel; ^10^The Drama and Health Science Lab and the Emili Sagol Creative Arts Therapies Research Center, University of Haifa, Haifa, Israel

**Keywords:** aging, playfulness, locus coereleus, noradrenalin (NA), arousal, exploration, uncertainty, drama therapy

## Abstract

Healthy cognitive aging emphasizes preserving cognitive functions essential for independence and well-being. Developing interventions that promote cognition and resilience in older individuals is crucial. Social playfulness, characterized by spontaneity and mutual enjoyment, allows individuals to step away from routine roles and engage in novel and surprising exchanges. Emerging evidence suggests that social playfulness is a promising approach for supporting cognitive functions in aging in a joyful and engaging way. In this theory and hypothesis manuscript, we propose a neurobiological pathway mediating the effects of social playfulness on cognition. Playful interactions generate high levels of uncertainty, requiring continuous adaptation and exploration. We suggest that these demands engage the locus coeruleus-noradrenaline (LC-NA) system, which is crucial for navigating uncertainty and sustaining arousal and flexibility needed to adapt to the dynamic and unpredictable nature of playful interactions. Importantly, the collaborative and safe environment of playfulness transforms this uncertainty-driven noradrenergic activation into an engaging and rewarding experience, enhancing focus, positive affect, and flexibility. In older adults, where LC-NA functionality may decline with age, social playfulness could counteract cognitive decline by upregulating this system. We review evidence linking LC-NA integrity to cognitive health and explore how playfulness might mitigate the deterioration of cognitive functioning by training executive functions and promoting novelty and exploration. This framework bridges neuroscience, cognitive psychology, and creative-arts therapies, highlighting social playfulness as a tool for healthy aging. We emphasize the need for further research to validate this hypothesis and explore its implications for designing interventions that leverage social playfulness to enhance cognitive resilience in older populations.

Social playfulness is a dynamic and engaging form of social interaction characterized by spontaneity, mutual enjoyment, and creativity, where individuals step away from routine roles and predictable patterns to engage in novel and often surprising exchanges (e.g., changing your voice to take on the role of a child) (Huizinga, 1949; Lieberman, 1977; Proyer, 2013). Social playfulness peaks during childhood—serving critical developmental purposes (Andersen et al., 2023; Proyer, 2013)—and continues to bring moments of joy and social connection in adulthood (Parker et al., 2023). While playful behaviors may diminish in frequency and spontaneity over the lifespan, they continue to hold significant value in later life (Andersen et al., 2023). Indeed, interventions incorporating social playfulness in older adults have demonstrated positive benefits, enhancing mental health and social connectedness (Bassis et al., 2023; Keisari et al., 2024b). Remarkably, the benefits of social playfulness in older adults extend beyond mental health to enhance cognitive performance and executive functions, as demonstrated by a series of studies from our lab (Keisari et al., 2022a; Keisari et al., 2024a; Benjamini et al., 2024). In this hypothesis and theory article, we delve into this less charted territory, focusing on the cognitive effects of social playfulness in older adults. Drawing from diverse fields—including art therapies, cognitive psychology, and neuroscience—we propose a theoretical framework for social playfulness and outline a hypothetical neurobiological pathway mediating its impact on cognition in later life.

The first three sections of this manuscript aim to synthesize literature from diverse fields, to offer a theoretical framework of social playfulness and to present a testable hypothesis on the neurobiological mechanisms through which social playfulness enhances cognition. Section 1 explores the concept of social playfulness, its pivotal role in human life and its cognitive benefits for older adults. Section 2 defines the core characteristics of playful interactions, i.e., unpredictability and reciprocity, and suggests how these features may contribute to cognitive enhancement. Section 3 introduces the locus coeruleus-noradrenaline (LC-NA) system as a key neurobiological mechanism, mediating the cognitive benefits of social playfulness, particularly in older adults. LC, a small yet vital brainstem nucleus, is known to exert broad effects on focus and cognition and to facilitate exploratory behavior under uncertainty (Aston-Jones and Cohen, 2005; Sara, 2009). Crucially, LC is believed to play a pivotal role in healthy cognitive aging (Mather, 2020; Poe et al., 2020; Sara, 2009).

Healthy aging emphasizes building and preserving the physical, cognitive, and social abilities needed to maintain well-being in later life (World Health Organization., 2020). The aim is not just to extend life but to “add life to years,” enabling older individuals to actively participate in families, communities, and societies. Therefore, it is crucial to develop interventions that not only provide training for cognitive functioning but also actively engage older adults in innovative, curious, and dynamic social activities (Sakaki et al., 2018). In this context, social playfulness emerges as a promising approach (Keisari et al., 2024b). In the concluding Section 4, we propose that engaging in exploratory social activities and stimulating the LC through social playfulness may offer a promising pathway to promote cognitive health and support healthy aging.

## 1 Background: social playfulness and its benefits in older age

*Johnny tries to impress Lisa with his famous pancakes for breakfast. When he opens the fridge, he exclaims “Oh no! This is a disaster! We’re out of milk!”. Lisa laughs, wraps herself in a light blanket and declares, “Superheroine is off on a mission to save her lover and bring him milk!” They look at each other, laugh, and hug.*

Play is at the heart of human life. It is a universal language that transcends cultures and generations, and that enables people to experience the world and create meaningful connections (Huizinga, 1949; Winnicott, 1971). Dutch historian Huizinga (1949) suggested that the essential quality of being human is reflected in the term *homo ludens* or “the individual as the player” as opposed to homo sapiens (“the knower or the wise”), since play is the starting point for all inquiry and exploration. Social playfulness, as an extension of this broader concept of play, refers to playful interactions between individuals that foster novelty, spontaneity and imagination.

In the following section, we will explore the progression from individual play to social play and ultimately to social playfulness, highlighting how play’s inherent spontaneity and creativity are enriched through social interactions and how these qualities distinguish social playfulness as an open, imaginative form of engagement. We will then review the benefits of structured social playfulness interventions in older adults, followed by an examination of its cognitive impacts within this unique population.

### 1.1 From play to social playfulness

Play is a voluntary, intrinsically motivated activity or behavior that exists outside of everyday reality and is often characterized by enjoyment, spontaneity, and creativity (Barnett, 2011; Huizinga, 1949). In his seminal work, Huizinga (1949) suggested that play exists beyond the bounds of ordinary reality, yet it maintains a connection to reality, such when a child in their bed pretends it is a boat, and they are a sailor navigating the ocean. Play consists of actions or thoughts, expressed in novel combinations, such as when a parent takes the role of a child. It is accompanied by a particular positive mood, during which the individual is more inclined to behave or think in a spontaneous and flexible way (Bateson and Martin, 2013). Play has no immediate utility beyond play itself, as opposed to rule-governed competitive sports, for example (Huizinga, 1949; Proyer, 2017). Research has shown that playing provides rich opportunities for development in all areas of life and serves important functions (Shen and Masek, 2023).

Play takes on new dimensions when shared with others. Winnicott underscored this idea in his memorable quote related to the importance of the other’s presence during the game of hide and seek: “It is a joy to be hidden, and disaster not to be found” (Winnicott, 1965, p. 186). This quote illustrates how a playful activity is validated and becomes meaningful by the mere presence of another person. Indeed, studies have indicated that the involvement of others enhances the positive effects of fun activities (Reis et al., 2017). Furthermore, social play significantly expands the scope of novel possibilities and explorative potential (Pellegrini, 2009). Importantly, social play is not a solely human endeavor. Rather, this energetic and rewarding activity is present in most mammalian species, and its contribution to the development of social, emotional and cognitive skills is widely recognized (Vanderschuren et al., 2016).

It is crucial to distinguish between social play and social playfulness in adulthood, as referred to in the current manuscript. Social play in adulthood often involves organized, rule-based activities with defined objectives, such as board games, card games, or digital games. In contrast, social playfulness represents an open, spontaneous, and imaginative attitude toward interaction, unbounded by rules or specific goals (Proyer, 2017; Shen, 2020). This playful quality transforms adults’ interactions into a freeform, explorative space, distinct from organized and rule-governed social play (Lieberman, 1977).

### 1.2 Social playfulness in older ages

While young children regularly engage in social playfulness, these spontaneous incidents may diminish in frequency or spontaneity over the lifespan (Andersen et al., 2023). As natural social playfulness decreases, structured interventions can offer older people opportunities to engage in playful interactions.

**FIGURE 1:**
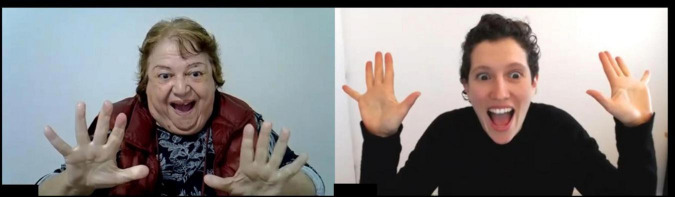
A moment captured during a playful interaction conducted over Zoom in one of our studies (Golland et al., 2024). This illustrates the engaging and joyful nature of social playfulness even when conducted in a virtual setting.

Indeed, social playfulness can be encouraged, practiced and learned (see for example Bassis et al., 2023; Keisari et al., 2020; Morse et al., 2018). One way to intentionally engage adults in social playfulness is through exercises derived from improvisation, which are the core of creative arts therapies, e.g., drama therapy, psychodrama and dance movement therapy (de Witte et al., 2021; Johnson, 2009; Sajnani, 2012) as well as the performance arts (Spolin, 1999). Examples include playback theater (improvisational theater based on personal stories, see Fox, 1999), improvisational theater groups (Felsman et al., 2023; Hainselin et al., 2018), and improvisational storytelling (Basting, 2006). As will be reviewed in this section, externally facilitated activities involving social playfulness with older adults have been consistently shown to have remarkable positive effects on social and psychological health indicators (Keisari et al., 2024b; Swinnen and De Medeiros, 2018). Supplementary Table 1 contains detailed description of studies exploring various forms of social playfulness interventions for older adults. Figure 1 demonstrates a fraction of a second taken from a virtual playful interaction between an older participant and a facilitator in one of our studies (Golland et al., 2024; Keisari et al., 2024a).

Studies of group interventions for older adults featuring playful activities have reported improvement in social connectedness, self-development, and a sense of flow, spontaneity, and tolerance of uncertainty (Bassis et al., 2023; Lindquist et al., 2021; Morse et al., 2018). A playback theater intervention with older adults based on improvisational play and sharing of personal stories found substantial enhancements in subjective and psychological wellbeing and a reduction in depressive symptoms (Elkarif et al., 2024; Keisari et al., 2022b). Participants reported that the playful interaction was enjoyable, strengthened their sense of competence, and promoted social connectedness (Keisari et al., 2020). Studies have shown that even a single short nonverbal playful interaction as compared to a matched control condition enhanced mood and social bonding (Keisari et al., 2022a), reduced loneliness, and increased salivary oxytocin levels in older age (Abu Elheja et al., 2021).

Interventions that involve playful interactions were also found to have positive effects on people with dementia (Hafford-Letchfield, 2013). Two major projects that involved playful storytelling for people with dementia demonstrated that creative playful verbal expressions facilitated communication, social engagement, interconnectivity, humor, and contentment (Basting, 2006; Swinnen and De Medeiros, 2018). Similarly, playful interventions that are based on improvisational theater have shown significant benefits for individuals with dementia by prioritizing present-moment engagement, spontaneity, and collaboration (Hafford-Letchfield, 2013). These interventions enhance positive affect, self-esteem, and cognitive stimulation while reducing depressive symptoms and fostering deeper social bonds (Stevens, 2012; Zeisel et al., 2018). Programs like the Memory Ensemble™ demonstrate how improve can challenge stigmas surrounding dementia, offering opportunities for creative expression and meaningful participation (Dunford et al., 2017). Similarly, social playfulness activities inspired by the Red Hat Society have been found to significantly predict resilience growth by fostering positive emotions and social interactions (Chang and Yarnal, 2018).

In sum, interventions that incorporate social playfulness have demonstrated notable benefits for older adults, enhancing psychological well-being, social connectedness, and adaptability. Across various studies, playful activities in group settings have been linked to improvements in mood, self-perception, and the capacity to tolerate uncertainty. These findings suggest that structured opportunities for social playfulness can help counteract the natural decline in spontaneous playfulness with age, fostering a sense of aliveness, as well as social and mental functions.

### 1.3 The effects of social playfulness on cognition in older age

The potential deterioration in cognitive functioning has emerged as one of the most concerning aspects of aging (Craik and Salthouse, 2011). The main domains of cognition which tend to decline with age include memory, reasoning abilities, processing speed, and various executive functions aspects, such as inhibition (Salthouse, 2019). Notably, research has indicated that an exploratory mindset, which is one of the characteristics of playfulness, is related to better cognitive functioning in older age (Sakaki et al., 2018). For instance, individuals who are characterized by a willingness to explore, tolerate, and engage with new and unfamiliar experiences, tend to exhibit less cognitive decline with age (Chapman et al., 2012; Williams et al., 2013; Ziegler et al., 2015). Studies have shown that that scripted theater classes, incorporating role-playing, dramatic activities, and rehearsal performances, enhance word recall, problem-solving abilities, and episodic memory in comparison to non-dramatic conditions (Noice et al., 2015; Noice and Noice, 2021). Inspired by these positive indicators, several studies in our lab have examined the effects of short playful interactions on cognitive performance in older age.

A cross-over randomized control trial involving 34 participants with a mean age of 84 demonstrated that a short session of the mirror-game, a common theater exercise based on dyadic synchronized movement incorporating playfulness and spontaneity, led to improved performance on the attention subscale of a cognitive screening test (The Montreal Cognitive Assessment: MoCA, Nasreddine et al., 2019) and faster detection of spoken words in noise compared to a physical activity control group (Keisari et al., 2022a). These improvements may have real-life implications for social interactions and daily activities (Heinrich et al., 2016). Another cross-over randomized control trial with a second group of 34 participants with a mean age of 85 examined the impact of a 15-mi dyadic playful interaction. This interaction involved the co-creation of a movement story by a participant and the drama therapist, which was compared to a control condition that consisted of personal conversations and an exercise class. The results indicated that the enhanced playful interaction as compared to the control condition led to improved performance on a digit span test, a standard clinical test for working memory capacity in older ages (WISC III), (Benjamini et al., 2024; Wechsler, 1991).

Finally, a recent cross-over randomized control trial in our lab involving 68 older adults with a mean age of 88 indicated that a short playful activity of 15–20 min led to higher scores on a digit span test and word fluency task, and faster response times on the Flanker test compared to a control condition of an exercise class. This improvement was observed in both online and face-to-face interactions (Golland et al., 2024; Keisari et al., 2024a).

## 2 Theoretical framework: core characteristics of social playfulness

Despite wide implementations of social playfulness as well as multiple research indications of its benefits discussed in the previous section, there are no well-defined theoretical frameworks of playfulness as a social behavior. Previous research has mainly studied playfulness as a predisposition or trait. For instance, Barnett (2007) defined playfulness as the predisposition to frame a situation in such a way as to provide oneself and others with amusement, humor, and entertainment. A more recent definition by Proyer (2017) described playfulness as an individual differences variable that allows people to frame or reframe everyday situations such that they experience them as entertaining, and/or intellectually stimulating, and/or personally interesting. Yarnal and Qian (2011) studied playfulness as a predisposition in older adults, and identified characteristics such as optimism, cheerfulness, amusement, positivity, enthusiasm, and relaxation, along with a tendency toward mischief, naughtiness, clowning, joking, and teasing. While these theories provide a useful basis for understanding the mindset of playfulness, they do not capture the dynamics of social playfulness as a particular behavior in context. One exception is a framework developed by Shen (2020) and Shen and Masek (2023) that relates to the specific conditions that encourage or hinder expressions of playful states. They suggested that even for individuals who naturally exhibit lower levels of spontaneous playfulness, engaging in playful states is possible in a safe, supportive environment that encourages playful activities.

What are playful social interactions? How are they different from ordinary ones? How can playful interactions improve cognition in aging? In the following section we provide a unified theoretical framework for social playfulness by defining two essential components of playful interactions, namely, (a) *unpredictability* and (b) *reciprocity*, which are presented in sections 2.1 and 2.2., respectively. We demonstrate how these two components are fundamental to human functioning and how they can contribute to cognition.

### 2.1 Unpredictability—being spontaneous and exploring novel moves

Social playfulness is inherently spontaneous and unpredictable, often defying preexisting scripts (Krueger et al., 2017; Schwenke et al., 2020). While playful interactions follow a basic framework of roles and actions, their progression unfolds dynamically, shaped by participants’ contributions and fuelled by the “Yes…and” principle (Bermant, 2013). Unlike routine interactions, which aim to minimize uncertainty (FeldmanHall and Shenhav, 2019; Lehmann et al., 2023), playful interactions thrive on amplifying novelty and surprise, stepping outside rehearsed patterns through imagination.

The uncertainty inherent to playful interactions promotes exploratory behaviors and fosters a “let’s try” mindset—an adaptive response to uncertainty, central to human decision-making (Friston, 2010; Whalen, 2007). The explorative nature of playfulness drives cognitive control mechanisms, necessary to navigate novel scenarios, inhibit habitual responses, allocate and sustain attention. Indeed, it has been repeatedly shown that creative exploratory processes are grounded in working memory (De Dreu et al., 2012; Kenett et al., 2023). These mechanisms are necessary for participants to balance the unpredictability of play with the need to maintain coherence and responsiveness during interactions.

Engaging with novelty has significant effects on cognition, including enhancing working memory encoding (Mayer et al., 2011), speeding up response times (Schomaker and Meeter, 2015), and improving perception (Schomaker and Meeter, 2012). Notably, processing novel stimuli, as opposed to familiar stimuli, is associated with better learning outcomes in both young and older adults (Axmacher et al., 2010; Bunzeck et al., 2012; Schomaker and Meeter, 2015). In sum, learning and cognition is enhanced when expectations are uncertain, outcomes are surprising, or contingencies are likely to change (Nassar et al., 2016; Axmacher et al., 2010; Yang and Tang, 2011).

While uncertainty in daily life often induces stress and avoidance (Carleton, 2016), playful interactions provide a safe and enjoyable environment where individuals can actively engage with uncertainty (De Wever et al., 2023). Thus, the collaborative nature of social playfulness creates an optimal environment for fostering an explorative mindset, driving curiosity and novelty-seeking, both of which are defined as intrinsic human drives to explore and grow (González-Cutre et al., 2016). For instance, improvisational theater, a form of playful interaction, has been shown to reduce intolerance of uncertainty and alleviate anxiety, particularly in younger populations (Felsman et al., 2020, 2023; Krueger et al., 2017). On a shorter timescale, we have systematically found elevated levels of positivity, enjoinment and flow, following short-term playful interactions (Benjamini et al., 2024; Keisari et al., 2022a; Keisari et al., 2024a).

To summarize the above, the spontaneous and collaborative nature of social playfulness is an ideal setting for navigating uncertainty and encouraging exploration. By reframing uncertainty as an enjoyable and rewarding experience, playful interactions enhance exploration and novelty-seeking—qualities that are deeply rooted in cognitive mechanisms such as attention, memory, and executive control. We propose that the cognitive demands of social playfulness, combined with its encouragement of an exploratory mindset and openness to new experiences, are central to its beneficial effects on learning and cognition. This makes social playfulness a promising approach for cognitive enhancement, particularly in older adults.

### 2.2 Reciprocity—co-creating with others

The second key component of playful interactions is their social nature, which requires continuous acts of mutual agreement and collaboration between participants (Pendzik, 2006). No action takes on its full meaning until it occurs and elicits response from others. This dynamic exchange allows participants to share and fuse their imaginative worlds, co-creating the meaning of the interaction (Kindler, 2010; Romanelli et al., 2017). Theories on improvisation suggest that these joint moments require continuous adaptation to social cues from others and are characterized by an increased sense of shared experience, where each individual action seems to be the right one and complement the actions of the partner (Sawyer, 2000; Seham, 2001). Indeed, studies have shown that the interdependence and reciprocity inherent in social playfulness can quickly enhance feelings of closeness among participants, even when they are strangers to one another (Keisari et al., 2022a; Keisari et al., 2024a).

During social interactions, uncertainties about individuals’ own future actions are compounded by uncertainty as to who the others are and how they might act at any given moment (Berkay and Jenkins, 2023; Lehmann et al., 2023). Each decision, from returning a smile to suggesting a conversational topic, relies on assumptions that may or may not hold true. Managing these uncertainties is critical for productivity, well-being, and social survival (FeldmanHall and Shenhav, 2019). Combining the spontaneous and unpredictable nature of social playfulness, discussed in the previous section, with the inherent uncertainty of social interactions underscores how social playfulness amplifies both uncertainty and the cognitive effort required to navigate it to an extreme degree. Unlike routine exchanges that aim to minimize ambiguity and follow familiar scripts, playful interactions thrive on unpredictability, driven by one’s own imaginative actions and the surprising responses of the partner (Sawyer, 2000; Seham, 2001). In the context of cognition, social playfulness extensively engages multiple processes of social cognition, including recursive mutual predictions, attentional focus, mentalizing, and real-time adaptation (Lehmann et al., 2023).

To summarize, playful interactions amplify uncertainty and sustain it throughout the interaction. The constant need to attend to and anticipate the partner’s actions stimulates mechanisms of social cognition, such as mentalizing, attention allocation and real-time adaptation. We propose that the engagement of these mechanisms during episodes of social playfulness contributes to its cognitive benefits, particularly in populations where cognitive functioning is compromised.

### 2.3 Co-creating in uncertainty: an example

A good example of the ways in which reciprocity and uncertainty intertwine and define playful interactions can be seen in a simple playful activity known as the “Continuous Story,” a collaborative storytelling activity in which participants (here Lisa and David) take turns contributing to the development of a story.^1^ For instance, Lisa says: “One sunny morning, Sarah woke up and decided that today was too beautiful to go to work…” This creates the setting for the story, and now David is free to take the story in any direction: Did she go to the beach? Did her boss call? David continued: “Sarah went to her favorite coffee shop on Maple Street.” Lisa accepted his idea and elaborated: “As she walked in, the aroma of freshly baked pastries filled the air, and she noticed her favorite table by the window was available.” David: “She ordered a cappuccino and a blueberry muffin, then settled down at the table with a book she had been eager to read.” Lisa added: “Just as she was getting into her book, an old friend from high school, Alex, walked in and waved at her enthusiastically.” David accepted this idea and took it even further: “Alex joined her at the table and handed Sarah an envelope, saying, ‘I found this in my attic today and thought you might want to see it.’ Inside was a photograph of them at their high school graduation, with a strange handwritten note from Sarah on the back, reading…” and the story continues to evolve.

The Continuous Story captures the unpredictable and spontaneous nature of playfulness, since each participant needs to build on the ideas put forward by another person, which form a narrative that constantly evolves in unexpected ways. At the same time, it demonstrates how playfulness is collaborative, in that it relies on the acceptance and incorporation of other’s ideas to create a narrative that is not only new but also shared. While playful interactions mimic regular social interactions to a certain extent, they significantly amplify them by frequently creating intense imaginative experiences characterized by a heightened sense of curiosity, alertness, and flow. However, the dynamics of this activity can shift significantly when a non-playful response arises. For example, if one participant were to criticize a contribution—saying, “That doesn’t make sense”—or outright reject an idea, the collaborative flow of the activity would be disrupted. These moments reveal the delicate balance required for playful interactions: they depend on an environment of mutual acceptance, where ideas are embraced, expanded upon, and celebrated rather than constrained or dismissed.

## 3 Locus coeruleus: the neurobiological mechanism mediating the effects of social playfulness on cognition

The previous sections suggested that social playfulness creates a dynamic environment, rich in uncertainty and demanding cognitive flexibility and adaptability. This environment mirrors the uncertainty inherent in many real-life situations, particularly those that require exploratory moves as well as rapid cognitive and emotional adjustments, such as adapting to a new workplace or professional role, becoming a parent, or responding to a tricky comment in social conversation. In this section we propose that these demands for flexibility and exploration recruit the noradrenergic cortical arousal system, known to play a key role in situations requiring exploring, learning and adapting in the context of uncertainty (Jordan, 2024).

The cortical arousal system originates in a small brainstem nucleus, the locus coeruleus (LC), which serves as the main source of noradrenaline (NA) in the brain (Mather, 2020; Poe et al., 2020). The LC receives various signals all related to arousal (e.g., wakefulness, sleep, stress) and sends NA projections that innervate almost every region of the brain and change the receptivity and excitability of the target regions. The LC-NA system is considered to be uniquely positioned to modulate alertness and exert a significant influence over global brain states, shaping cognitive processes, and promoting behavioral adaptations (Aston-Jones and Cohen, 2005; Berridge and Waterhouse, 2003; Mather et al., 2016; Sara, 2009).

### 3.1 LC-NA activation is linked with uncertainty and exploratory behavior

Of relevance for its function in social playfulness, the LC-NA system plays a unique role when facing situations with high levels of uncertainty requiring novel, exploratory behaviors (Yu and Dayan, 2005). Theoretical models of LC functions suggest that in the context of uncertainty the LC-NA may serve as an alarm system for contextual switches by enhancing the saliency of sensory-induced signals, suppressing top-down expectation-driven information, and promoting new learning about the context (Aston-Jones et al., 2000; Gu, 2002; Poe et al., 2020). Accordingly, research applying both central and peripheral measures of NA have shown that LC-NA activity increases with higher levels of unpredictability and unexpected external changes (Aston-Jones et al., 2000; Poe et al., 2020; Sara and Bouret, 2012; Vankov et al., 1995). Furthermore, a broad array of studies linked the activation of the LC-NA system with increase in exploratory behaviors that are needed during states of high uncertainty when well-trained models of behavior are less productive (such as during social encounters in a foreign country). High levels of NA increase the probability of strategy shifts, whereas low levels facilitate perseverative behavior (Yu and Dayan, 2005). Complementary animal research has shown that increasing NA levels leads to more value-free-random-like exploratory behavior (Tervo et al., 2014), whereas reducing NA increases choice consistency (Jahn et al., 2018). Recent pharmacological studies in humans have indicated that value-free random exploration is attenuated under the influence of an NA antagonist during decision-making tasks (Cremer et al., 2023; Dubois et al., 2021). It has been suggested that NA may exert these effects by acting as a “reset button” that interrupts ongoing information processing, thereby inhibiting the use of previously accumulated knowledge in favor of exploring new options (Cremer et al., 2023).

As discussed in section 2, high levels of uncertainty are a defining characteristic of social playfulness, where an imaginary world is spontaneously co-created, often taking surprising turns and incorporating unpracticed social dynamics. Accordingly, we propose that playful interactions engage the LC-NA system, enabling players to adapt to continuously unfolding scenarios and explore novel responses. In support, the key role of the LC-NA has been linked with mind states related to playfulness, including curiosity (Monosov, 2024; Sakaki et al., 2018), fluid (as opposed to crystallized) intelligence (Tsukahara and Engle, 2021) and flow (Van Der Linden et al., 2021).

Notably, animal studies have shown that pharmacologically manipulating noradrenaline (NA) levels can affect social play behavior (Achterberg and Vanderschuren, 2023; Siviy and Panksepp, 2011; Vanderschuren et al., 2016). Specifically, alpha-1 adrenergic antagonists, beta-adrenergic antagonists and alpha-2 adrenergic agonists reduce social play behavior (Beatty et al., 1984; Normansell and Panksepp, 1985; Siviy and Baliko, 2000). Drugs that reduce noradrenaline reuptake also reduce rodent social play behavior via alpha-2 adrenergic receptor action (Vanderschuren et al., 2008). Consistent with alpha-2 adrenergic receptor activity reducing social play, an alpha-2 antagonist can increase social play in rodents (Siviy and Baliko, 2000). The opposing effects of different adrenergic receptors on social play makes sense; alpha-2A adrenergic receptors are generally inhibitory, including inhibiting the locus coeruleus itself via autoreceptor activity (Callado and Stamford, 1999), whereas beta-adrenergic receptors (and sometimes alpha-1 receptors) tend to be excitatory (Farzam et al., 2024). While these pharmacological approaches in rodents highlight the role of the LC-NA system in playfulness, they do not address the question of the downstream effects of engaging in playfulness, which is the focus of the current manuscript.

It is important to note that social playfulness is a complex processes, involving multiple neurobiological mechanisms and agents (Achterberg and Vanderschuren, 2023; Siviy and Panksepp, 2011), which lie beyond the scope of this manuscript. As specified in the following sections, here we highlight the pivotal role of the LC-NA system in mediating the beneficial effects of playfulness on cognition, by linking cognitive performance with arousal.

### 3.2 The LC-NA system modulates arousal and cognition

Models of LC-NA function help to further decipher how the uncertainty, inherent to social playfulness, enhance cognition through elevated arousal (Mather and Harley, 2016; Sara, 2009). When predictability is low, people need to be prepared for a plethora of possible scenarios, which requires elevated alertness and body’s metabolic resources (King and Williams, 2009). Studies have suggested that the brain broadcasts signals of uncertainty across widespread neural circuits via low-level arousal systems such as LC-NA which is capable of profoundly shaping the global states of the brain through the modulatory action of NA (Jordan, 2024; Yu and Dayan, 2005).

Accordingly, the recruitment of the LC-NA system increases body arousal and, in addition, promotes an efficient and appropriate cognitive response through its widespread modulation of cortical dynamics (Sara and Bouret, 2012). Specifically, it has been suggested that noradrenaline released in forebrain structures may facilitate sensory processing and enhance cognitive flexibility and executive functions (Sara, 2009). In support, previously theoretical frameworks have identified the LC-NA system as playing key roles in focused attention and attentional control (Aston-Jones et al., 2000; Aston-Jones and Cohen, 2005; Mather, 2020; Sara, 2009; Unsworth and Robison, 2017; Yu and Dayan, 2005). Abundant empirical research demonstrated the effects of specific noradrenergic manipulations on various cognitive functions, including attention, working memory, cognitive flexibility, response inhibition and emotional memory (see Chamberlain and Robbins, 2013 for a review).

We suggest that the high levels of uncertainty, instantiated by the spontaneous, unpredictable and reciprocal playful exchanges, necessitate the recruitment of metabolic resources, peripheral arousal, and the activation of the brain’s LC-NA system to adapt and navigate uncertainty. In support for this suggestion, we have found that episodes of playful interactions induced increases in self-reported subjective arousal in older adults (Golland et al., 2024) and young children (Yaffe et al., under revision^2^), and these increases were significantly larger than following episodes of ordinary interactions. While little research has been done on peripheral nervous system functions in playful and creative contexts, a few studies have indicated that playful interactions elicit cardiovascular and subjective arousal (Armstrong et al., 2015; Noy et al., 2015) and there are some indications for increased sympathetic activity during nonsocial creative processes, such as divergent thinking (Khalil et al., 2023; Silvia et al., 2014) and states of flow (de Manzano, 2010).

As detailed in section 4, we hypothesize that in older individuals, where age-related deterioration in LC-NA functioning may occur (Mather, 2020), the upregulation of LC-NA activation through playfulness mediates the observed improvements in cognitive performance following playful episodes (Keisari et al., 2022a; Keisari et al., 2024a; Morse et al., 2018).

### 3.3 The optimal positive arousal hypothesis

Notably, not all increases in arousal benefit cognition. For instance, the well-known Yerkes-Dodson law suggests that optimal performance occurs at moderate arousal levels (Yerkes and Dodson, 1908). Recent research supports this inverted-U-shaped relationship, demonstrating that peak decision-making performance is achieved at moderate arousal, while high levels are suboptimal for performance (Beerendonk et al., 2024). In line with that suggestion, stress was shown to disrupt active cognition by impairing working memory (Qin et al., 2009; Schoofs et al., 2008, 2009) and cognitive flexibility (Plessow et al., 2011, 2012; Sänger et al., 2014).

Research on LC-driven states of arousal provides the neurobiological framework for the inverted-U-shaped relationship with cognition (Aston-Jones and Cohen, 2005). Specifically, literature suggests that the NA-driven cognitive enhancement, reviewed in the previous sections, is grounded in a mid-range of LC-NA activity, where there is an optimal balance between tonic (background levels of NA release) and phasic (quick bursts of NA) LC activity (Aston-Jones and Cohen, 2005; Poe et al., 2020). Accordingly, low levels of tonic LC activity leads to inattention and poor performance, whereas an increase to an intermediate range sharpens attention and enhances behavioral performance. When an optimal state of tonic activity is coupled with maximal phasic activity (thus increasing the signal-to-noise ratio), there is high responsivity to relevant sensory stimuli and more focused attention on task-related, novel, or unpredictable stimuli. Excessive tonic LC activity leads to lowered LC phasic activity, hyperarousal, task disengagement and reduced performance (Wainstein et al., 2022). In fact, such heightened LC activity is considered to play a significant role in anxiety (Morris et al., 2020), mediating many of its symptoms (Ross and Van Bockstaele, 2021), as well as the hypervigilant responses in posttraumatic stress disorder (Naegeli et al., 2018). This suggests that overly high levels of arousal and corresponding tonic LC-NA activity may impair cognitive performance and lead to emotional negativity and anxiousness.

As discussed in previous sections, social playfulness thrives on uncertainty. While high levels of uncertainty could lead to stress and anxiety (Carleton, 2016), studies consistently demonstrate that playful interactions elevate mood and foster positive mental states (Bassis et al., 2023; Morse et al., 2018; Zeisel et al., 2018). A key part of our hypothesis suggests that social playfulness holds a unique balance between the stimulating challenge of uncertainty and the protective effects of psychological safety and connectedness. The unpredictable and reciprocal nature of playful interactions compel individuals to continuously read and adapt to their partners’ signals in order to plan the next move and reduce uncertainty (Sebanz et al., 2006; Wise et al., 2023). Notably, states of increased connectedness, occurring in cooperative settings, have been shown to foster positive social states, such as bonding, intimacy, and trust (Marsh et al., 2009; Mogan et al., 2017).

In line with this, one of the most well-documented effects of social playfulness, as reviewed in section 1.2, is its ability to significantly enhance feelings of connection and positive mental states. These include closeness, empathy (Ballon et al., 2007), elevated mood, and a strong sense of bonding (Bassis et al., 2023; Keisari et al., 2024b). Our research supports these findings, demonstrating that even brief playful interactions increased social connectedness, responsiveness, and positive affect in both older adults (Golland et al., 2024; Keisari et al., 2020) and young children (see text footnote 2).

**FIGURE 2:**
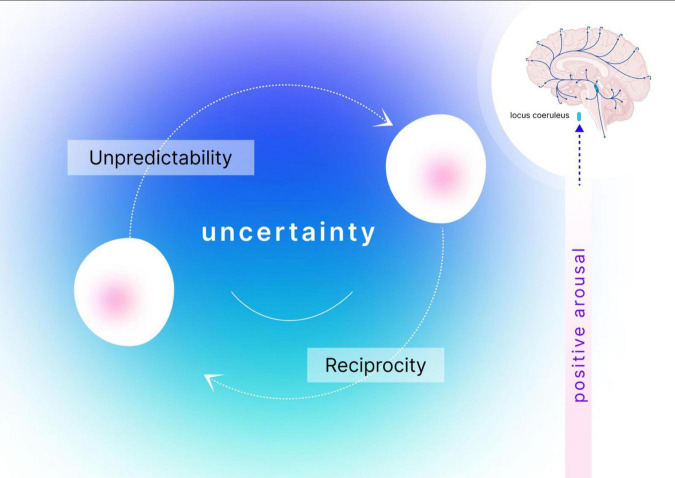
Illustration of optimal positive arousal hypothesis. Playful interactions are characterized by high levels of uncertainty, where each move is unpredictable, surprising, and critically influenced by the partner’s previous response. Navigating this social uncertainty activates the LCs-NA system, which plays a vital role in sustaining an explorative mindset and adapting to rapid changes and novel scenarios. Within the collaborative and safe environment of playful interactions, this noradrenergic activation drives significant increases in positive arousal, enhancing focus and engagement.

Integrating the above, we propose the optimal positive hypothesis of social playfulness, suggesting that playful interactions encourage individuals to explore and engage in novel scripts and behaviors. These interactions, marked by frequent shifts of narrative, high levels of novelty, and continuous adaptation to partners’ signals, require heightened alertness and flexibility. To meet these demands, individuals recruit the LC-NA system, which, in turn, enhances arousal, focus, and adaptability. At the same time, the strong reciprocity and cooperative nature of play, infused with positivity and a “yes, and” mindset, cultivate intimacy and psychological safety. This sense of security transforms the radical uncertainty and ambiguity of playful interactions into a catalyst for exploration and cognitive enhancement, rather than a source of stress and fear. In support, social security was found to promote novelty-seeking, curiosity, and cognitive openness (Carnelley and Ruscher, 2000; Melen et al., 2017; Stevenson et al., 2017), liking of novel stimuli, and willingness to learn (Green and Campbell, 2000; Luke et al., 2012; Melen et al., 2017; Mikulincer and Shaver, 2020), as well as enhanced sensory processing in younger and older adults (Nagar et al., 2022). We illustrate the main components of the Optimal Positive Arousal Hypothesis in Figure 2.

## 4 Implications for older age

Aging is generally defined as a multifactorial process, resulting in both biological and psychological changes (Hsu and Jones, 2012). The multisystem challenges of aging are well-documented, with cognitive decline at the forefront of older adults’ concerns (Salthouse, 2019). In this section we present evidence that aging is characterized by negative changes in novelty seeking (Spreng and Turner, 2021), as well as structural changes in the LC-NA system (Braak et al., 2011; Mather and Harley, 2016). Centrally, these changes were linked with decrease in mental health and cognition and enhanced chance for Alzheimer. At the last part of this section, we suggest that purposefully engaging in playful interactions may possibly address these age-related changes, by instigating exploratory behaviors, training executive functions, recruiting the LC-NA system and thus potentially preserving and enhancing cognition in older age.

### 4.1 Age-related changes in LC-NA

Abundant research suggests that LC plays a pivotal role in maintaining cognitive resilience in older adults and is related to the early pathological changes associated with Alzheimer’s disease (Mather and Harley, 2016).

The hallmark features of Alzheimer’s disease (AD) are tau tangles and amyloid plaques. Postmortem studies reveal that tau pathology begins in the locus coeruleus (LC) during a pretangle phase before spreading to other brain regions involved in memory (Braak et al., 2011). Furthermore, recent evidence suggests that the earliest phases of brain degeneration linked with Alzheimer’s disease are already mapped to the LC (Galgani and Giorgi, 2023; Jacobs et al., 2021). As we do not yet have in vivo measures of LC pretangle tau, we cannot know how these early phases of Alzheimer’s pathology affect cognition. However, multiple findings demonstrate that LC structural decline later in life is associated with poorer cognition. For instance, older adults who died with relatively lower LC cell density declined faster cognitively prior to death (Wilson et al., 2013).

Advances in magnetic resonance imaging (MRI) allow detection of LC-specific contrast in regions corresponding to noradrenergic LC cells (Keren et al., 2015). These techniques have generated considerable evidence indicating that a higher LC MRI contrast is associated with better cognition in older adults (Clewett et al., 2016; Dahl et al., 2019; Liu et al., 2020). Specifically, a higher LC MRI contrast was shown to be associated with better episodic memory (Dahl et al., 2019, 2023; Hämmerer et al., 2018), higher subjective cognition (Bell et al., 2023), and reduced risk of developing mild cognitive impairment (Elman et al., 2021).

LC responsiveness to novelty appears to decline with increasing AD-related tau pathology. For example, functional MRI studies show that reduced LC activation to novel stimuli correlates with greater tau pathology and cognitive decline (Prokopiou et al., 2023). Animal studies further highlight the LC’s protective role: in a rat model of LC pretangle pathology, phasic LC stimulation (mimicking novelty responses) prevented memory decline and limited tau spread, while tonic stimulation or no stimulation did not (Omoluabi et al., 2021). These findings suggest that intermittent LC bursts tied to novelty may counteract the harmful effects of tau pathology.

Dahl et al. investigated the link between arousal, LC-NA activity and selective attention in younger and older adults (Dahl et al., 2020). They found that older adults displayed reduced noradrenergic responsiveness compared to younger adults, indicated by blunted pupil dilation, a non-invasive proxy for LC activity, and EEG responses. Crucially, they showed that both younger and older adults with a more responsive noradrenergic system performed better on attention tasks. This finding suggests that a robust and responsive LC-NA system supports attentional abilities throughout the lifespan, and its decline may contribute to the attentional difficulties experienced by some older adults.

Overall, these animal and human studies indicate that maintaining LC integrity in aging may help cognition in two ways. NA modulates cognitive processes such as episodic memory, working memory, and inhibition of irrelevant information. Impairments in the LC–NA system is thus likely to disrupt these cognitive processes. In addition, the LC–NA may contribute indirectly to cognitive function. It has long been observed that factors such as social engagement and learning seem to protect against cognitive impairment even when Alzheimer’s disease neuropathology is present in the brain (Stern, 2012). The emerging findings on the LC–NA system in aging and dementia suggest that this system may support these “cognitive reserve” effects (Robertson, 2013). We therefore suggest that cognitive interventions that involve exposure to new and stimulating experiences, such as learning new skills, engaging in complex problem-solving, and social playfulness may help maintain LC function and slow cognitive decline (Mather and Harley, 2016).

### 4.2 Age-related changes in exploration and novelty-seeking

As adults age, cognitive processes shift significantly. Adaptive control processes that support rapid and flexible thinking decline, while accumulated knowledge about oneself and the world grows (Spreng and Turner, 2021). This trajectory in cognitive aging encourages an exploitative mental mode, where the system tends to rely on existing prior knowledge to compensate for age-related sensory and cognitive changes. Accordingly, the levels of novelty-seeking, exploratory behaviors and novel choices significantly drop with age (Spreng and Turner, 2021) and multiple exploration-related traits, motivations and behaviors tend to decline (Costa et al., 2000; McCrae et al., 1999; Ziegler et al., 2015). Prominent examples are reductions in curiosity, a key driver of novelty-seeking behavior (Monosov, 2024; Sakaki et al., 2018), openness to experience, i.e., a person’s willingness to explore new ideas and experiences (Brodaty et al., 2010; Giambra et al., 1992; Kashdan and Silvia, 2009), variety seeking (Novak and Mather, 2007) as well as sensation- seeking, i.e., the tendency to pursue novel, varied, and intense experiences (Lawton et al., 1992). Animal studies mirror these findings, by showing that older animals demonstrate reduced exploratory behavior in novel situations (Collier et al., 2004; Dellu et al., 1994). A recent study showed that the extent of this exploitation bias in older adulthood is associated with lower microstructural integrity of the locus coeruleus (Turner et al., 2024).

Crucially, increased responsiveness to novelty in older age has been linked with better wellbeing, physical and mental health (Ferguson and Bibby, 2012) as well as performance in cognitive tasks involving learning, attention and memory (Bunzeck et al., 2012; Daffner et al., 2006). It has been previously proposed by others that practicing novelty and exploration may protect against age-related cognitive decline and promote adaptive aging (Ferreira et al., 2015; Sakaki et al., 2018). In the next section, we propose that social playfulness can act as a safe environment for maneuvering social novelty, uncertainty and surprise, training the related cognitive functions and neurobiological mechanisms, and potentially promoting healthy cognitive aging.

### 4.3 Social playfulness can facilitate better aging by training the LC-NA system and maintaining explorative and open mindset

As described in the previous two sections, aging is characterized by cognitive and neurological changes, including a decline in adaptive control processes that impacts daily functioning (Salthouse, 2019). Research highlights that these changes, in particular the decline in openness and sensation- seeking, contribute to decreased motivation for new experiences, which in turn is associated with a decrease in cognitive functions (Kashdan et al., 2009; Lawton et al., 1992; Sakaki et al., 2018). These reductions in exploration in aging are closely related to neurological alterations, especially involving the locus coeruleus-noradrenergic system (Mather, 2020; Mather and Harley, 2016). Furthermore, evidence links LC-NA integrity to improved cognitive functions in older age, as well as a lower risk of developing mild cognitive impairment and the early stages of Alzheimer’s pathology (Dahl et al., 2019, 2023; Hämmerer et al., 2018). While further research is needed to establish the optimal types and timing of interventions, the literature suggests that promoting safe exploration and novelty, potentially by stimulating the LC-NA system, holds significant promise for preserving cognitive health and promoting successful aging (Aston-Jones and Cohen, 2005; Daffner et al., 2006; Mather and Harley, 2016; Sara and Bouret, 2012).

At the outset of this paper, we introduced the core principles of playful social interactions—unpredictability and reciprocity—and highlighted how they instigate novelty and exploration in a safe setting (Huizinga, 1949; Shen and Masek, 2023; Winnicott, 1971). These interactions, characterized by spontaneous and reciprocal engagement, encourage creativity and adaptability in a positive and accepting environment (Felsman et al., 2020; Hainselin et al., 2018; Sawyer, 2000). This form of playfulness can serve as a potent tool for engaging the cognitive processes that are most susceptible to decline with aging (Keisari et al., 2022a; Keisari et al., 2024a). Specifically, engagement in uncertain and reciprocal activities can stimulate executive functions such as working memory, attention flexibility, and inhibition that typically decline in older age and that are linked with LC function (Mather and Harley, 2016). Building on the observed short-term effects of playfulness, we hypothesize that long-term interventions centered on social playfulness could provide older adults with cognitive training targeting these critical functions. Furthermore, if, as proposed in this manuscript, social playfulness engages the LC-NA network, a continuous practice in such interactions could not only mitigate cognitive decline but also enhance cognitive and neural resilience, thereby promoting healthier aging.

Finally, playful interactions are inherently social, relying on collaboration between participants (Bermant, 2013). The deep reciprocity embedded in social playfulness was found to foster a sense of closeness and belonging among older adults (Keisari et al., 2020; Morse et al., 2018; Woslov et al., 2024). In this context, social playfulness may represent a viable intervention to address the social isolation and loneliness experienced during aging, a condition strongly associated with cognitive decline and compromised mental health (Cudjoe et al., 2020; Shankar, 2017).

## 5 Future directions

The proposed framework of LC-driven optimal positive arousal in social playfulness and its links to cognitive functioning in older adults synthesizes findings from diverse literature and warrants empirical testing. First, the optimal positive arousal hypothesis can be evaluated using short, lab-adapted playful interactions. Neural mechanisms, particularly LC involvement, can be assessed using imaging techniques alongside peripheral measures like pupillometry (Clewett et al., 2018; Fan et al., 2023). These metrics, combined with markers of sympathetic activity and subjective reports of arousal and mood, could determine whether playful interactions—compared to more predictable control interactions—elicit heightened arousal and whether this arousal mediates cognitive improvements immediately following such episodes. Furthermore, indices of phasic and tonic components of LC activity, as well as of pupillometry and galvanic skin responses may indicate whether playful interactions indeed induce both tonic and phasic increases, as suggested by models of optimal arousal (Aston-Jones and Cohen, 2005; Nieuwenhuis et al., 2011). Additionally, controlled lab experiments manipulating levels of cooperation and support in playful interactions could explore whether psychological security is necessary to buffer the potential stress of unpredictability and whether heightened negative arousal impairs subsequent cognitive performance. Centrally to the current manuscript, examining age-related sensitivity to these factors can deepen our understanding of how playfulness supports cognitive functioning in older adults, potentially revealing age-specific mechanisms.

The demonstrated short term effects on cognitive performance in older adults (Benjamini et al., 2024; Golland et al., 2024; Keisari et al., 2022a; Keisari et al., 2024a), alongside the potential to enhance LC neural integrity and excitability through playfulness training (Mather and Harley, 2016), underscore the urgent need for further research into the long-term impacts of social playful interventions in aging populations. Longitudinal studies could determine whether such interventions yield enduring benefits for cognitive functioning. Moreover, future research should adopt dose-response designs to identify the optimal frequency and intensity of playful interactions necessary to maximize cognitive and emotional gains, providing actionable guidelines for intervention development.

In addition to its theoretical significance, this paper underscores the practical and implementational potential of social playfulness as an intervention for enhancing cognitive health and resilience in older adults. Playful interactions provide a natural and ecological approach that leverages the inherent human tendency to engage in play (Feniger-Schaal et al., 2024; Proyer, 2013; Shen, 2020; Shen and Masek, 2023). Unlike computer-based training programs, these activities are holistic, enjoyable, cost effective and intrinsically motivating, making them particularly suitable for older populations. These simple and accessible practices can be seamlessly incorporated into routine care settings, such as community centers, assisted living facilities, or home environments, offering a practical and scalable means of cognitive stimulation (see for example Bassis et al., 2023; Keisari et al., 2020; Morse et al., 2018). Lifestyle-focused research should further explore how the core elements of social playfulness—spontaneity, exploration, and connectedness—can be integrated into daily routines. Encouraging older adults to adopt social playfulness in their everyday lives has the potential to enhance both cognitive and psychological well-being, demonstrating the wide-reaching applicability of this approach. These research directions will not only strengthen the theoretical framework but also highlight the practical value of social playfulness in promoting healthy aging.

## Data Availability

Publicly available datasets were analyzed in this study. This data can be found here: no data is included.

## Footnotes

1 The example presented here is purely fictional and was created solely for the purpose of illustrating this discussion.

2 Yaffe, D., Shtoots, L., Reuveni, O., Keisari, S., and Golland, Y. (under revision). Short playful interaction improves executive functions in children. *Sci. Rep.*
