# Supplementary material for: Playful brains: a possible neurobiological pathway to cognitive health in aging
Source: Front Hum Neurosci. 2025 Feb 7;19:1490864. doi: 10.3389/fnhum.2025.1490864 (PMC11842457; doi:10.3389/fnhum.2025.1490864)
Supplement: Supplementary file 1 [file Data_Sheet_1.pdf]

Supplementary Materials 1.

Studies on the Implications of Social Playfulness for Older Adults

Category	Authors	Intervention Type	Intervention Description	Study Type	Sample Characteristics	Key Findings
**Improv**	(Zeisel et al., 2018)	Scripted-IMPROV: interactive improvisational drama	A semi-improvised drama intervention specifically designed for persons with dementia (PWD). Facilitators guided participants to act out roles in loosely scripted plays with interactive props and costumes to enhance engagement and affect.	A single-group pre–post study design	178 participants across 12 sites (adult day centers, assisted living facilities, and nursing homes); mean age 85.5 years. Diagnosed with dementia (MMSE ≤ 20).	Positive engagement and affect significantly increased, negative engagement decreased, and depressive symptoms reduced in participants initially classified as clinically depressed. No overall change in long-term quality of life.
**Improv**	(Morse et al., 2018)	Improvisational comedy course - Humor Doesn’t Retire Program	A six-week structured course in improvisational comedy for older adults, including activities such as role-playing, collaborative storytelling, and "yes, and..." exercises. The course emphasized active	Qualitative study	10 participants aged 57–81 (mean: 71.6 years); majority female.	Participants reported several perceived benefits, including increased positivity, a greater sense of comfort with the unexpected, enhanced self-awareness, and feeling accepted within their social groups. These benefits contributed to behavioral changes such as improved problem-solving abilities, greater ease in

			participation, fostering creativity, humor, and adaptability to unexpected			social situations, and the tangible outcome of an expanded, closer-knit social circle.
**Improv**	(Lindquist et al., 2021)	Improvisational comedy course - Humor Doesn't Retire Program	An 8-week course entitled "Humor Doesn't Retire," focused on improv techniques such as "yes, and…," building ensemble, object work, and minimizing physical movement for accessibility.	Mixed methods study	15 older adults (mean age 83.6 years), majority female, living in a long-term care facility.	Significant improvements in social isolation and perceived stress. Participants also reported improved spontaneity, listening skills, and communication.
**Improv**	(Yamamoto, 2020)	improvisational theater course for older adults	A 14-week course introducing older adults (ages 55+) to improvisation principles through exercises such as "yes, and…," short-form theater games, and role-playing activities. The course emphasized trust, acceptance, and presence, creating a supportive environment where participants engaged in playful and creative expression.	Qualitative study	6 women aged 65–80, with varied levels of experience (e.g., acting background or no prior training). All participants were part of a senior program at a community college.	Participants reported enhanced joy, mental stimulation, and social connection. They valued the opportunity to "play" and described the course as a tool for combating aging stereotypes and fostering community.
**Improv**	(Bassis et al., 2022)	improvisational theater course for older adults	A six-week course involving two weekly one-hour sessions in improvisational theatre. Activities included "Yes, and…" exercises,	A single-group pre–post study design	45 older adults (ages 67–92), 71% female, with no prior theater	Improvement in spontaneity, flow, and playfulness. No significant changes in cognitive flexibility were observed.

			storytelling, and object improvisation using props to enhance spontaneity, creativity, and flow.		experience. Conducted in daycare centers and retirement homes in Israel.	
**Improv**	(Sutherland et al., 2024)	Acting and Improvisation Course	A 12-week acting and improvisation course facilitated by a trained theater professional. Conducted in federally subsidized housing for older adults, sessions included improvisation exercises, role-playing, breathing techniques, and a culminating public performance.	Mixed methods study	14 African American older adults (ages 52–71), living in low-income housing. Majority female with moderate physical impairments.	Quantitative measures showed no significant changes in social connection. Qualitative data identified participants' motivations to enroll, such as meeting others, reducing shyness, and learning new skills.
**Improv**	(Stevens, 2012)	Stand-up Comedy and Improvisation Workshops	Eight weekly, two-hour workshops teaching people with mild dementia stand-up comedy and improvisation skills, culminating in a public performance. Activities included warm-ups, role-playing, and simple	Qualitative study	15 participants with mild dementia (ages 78–86), 8 men and 7 women, living in the community with carers.	Participants developed skills in comedy, displayed improved self-esteem, sociability, and communication. Carers noted increased memory and anticipation for sessions.

			scenarios to create and perform humor.			
**Improv**	(Hafford-Letchfield, 2013)	Improvisation Workshop for Older Adults with Dementia	A series of four weekly workshops (2–3 hours each) incorporating improvisation and comedic storytelling. Participants collaboratively created scenarios, culminating in a 30-minute "mockumentary" about a fictional visit by the Queen to their day center. Themes were participant-driven and emphasized creativity, engagement, and humor.	Qualitative study	12 older people with dementia, 4 family carers, 8 staff members, and 2 comedy trainers in a UK day center.	Enhanced participant engagement, laughter, sociability, and self-esteem. Staff gained insights into participants' life histories and strengths.
**Improv**	(Dunford et al., 2017)	Improvisational theatre workshop for early-stage Alzheimer's disease	The Memory Ensemble provided 6–9 weekly 90-minute sessions incorporating structured and flexible improvisational activities, such as "Yes, and..." exercises, metaphor-based emotional check-ins, and creative scenarios to enhance creativity and connection.	A case study	Persons with early-stage Alzheimer's disease and related dementias; small group sessions (6–14 participants and two facilitators).	Participants reported increased creativity, reduced anxiety, and a sense of community. The program fostered relational engagement and self-esteem.
**Playback theatre and drama therapy**	(Keisari et al., 2018, 2020;	Life-Review Playback Theater: A group	Playback theater involves participants sharing personal stories, which are	A mixed methods randomized controlled trial	78 community-dwelling older	The quantitative findings revealed significant improvements in mental health indicators, including self-

	Keisari, Palgi, et al., 2022; Keisari et al., 2023)	intervention combining life review principles with playback theater improvisation.	then enacted through theatrical improvisation by the group members. The intervention integrated life review therapy and playback theater.		adults (mean age: 79.6 years) attending adult day centers in Israel. Participants were split into experimental (*n*=40) and control (*n*=38) groups. The qualitative part of this research project included 27 older participants, and 13 staff members.	acceptance, personal growth, and satisfaction with life, alongside a reduction in depressive symptoms. These positive effects were sustained three months post-intervention. Participants and staff members reported spontaneous dramatic improvisation as an enjoyable activity that enriched the group experience. Dramatically enacting the life stories of others fostered a sense of purpose, contribution, and having a valuable role within the community. The program provided participants with opportunities for creative expression, strengthened their sense of social connectedness within the group, and the broader adult day center community. Additionally, the intervention facilitated a more integrative processing of life stories, enabling participants to address unresolved issues from their past.
**Playback theatre and drama therapy**	(Elkarif et al., 2024)	Tele-drama therapy: Group drama therapy conducted via an online platform	Playback theater involves participants sharing personal stories, which are then enacted through theatrical improvisation by group members. The intervention combined life review therapy with playback theater, focusing on the exploration of	Randomized controlled trial	111 older adults (ages 63–102) with constricted life-space mobility, divided into experimental (*n*=63) and control	Significant improvements in social connectedness, personal growth, and psychological well-being. Reduction in depressive symptoms. Loneliness did not significantly decrease, potentially due to participants' life circumstances.

			positive memories and future perspectives through theatrical improvisation in an online setting.		groups (*n*=63).	
**Playback theater**	(Woslov et al., 2024)	Playback theatre for Older Women	Long-term group sessions involving improvisational theatre based on participants' personal stories	Qualitative study	17 women aged 62–81, participants in community playback theatre groups for at least six months.	Participants reported personal growth, enhanced self-expression, and a positive connection to their ageing bodies. The intervention fostered playfulness, improved social bonds, and promoted emotional openness
**Playback theater**	(Chung et al., 2018)	Playback theatre	Playback theater involves participants sharing personal stories, which are then enacted through theatrical improvisation by group members. Six weekly sessions in total (about 1.5 hours, once a week	A single-group pre–post study design	18 older adults, mean age = 64.2	Participants experienced a significant improvement in their emotional well-being post intervention. However, there were no significant changes in participants' cognitive function or health-related quality of life.
**Theatre based intervention**	(Noice & Noice, 2013; Noice et al., 2015; Noice & Noice, 2021)	Participatory theater training	Participants engaged in script-based and improvisational acting exercises, led by professional acting instructors. The intervention consisted of twice-weekly 70-min sessions for 4 weeks.	The study employed a randomized controlled trial (RCT). Participants were assigned to either the theater intervention group or a waiting-list control group. Assessments included pre- and	A total of 115 participants (ages 68–94) in retirement homes	The theater intervention group demonstrated significant improvements in verbal fluency, immediate and delayed word recall, and observed tasks of daily Living compared to the control group. These findings suggest that participatory theater can be a meaningful tool for promoting healthy aging and enhancing cognitive functioning in older adults.

			** Note, this program emphasized cognitive engagement and memory recall through theatrical techniques and rehearsing performance.	post-intervention measures of cognitive functioning, psychological well-being, and other related outcomes.		
**Theatre based intervention**	(Yuen et al., 2011)	Participatory theater training	The "Seasoned Arts at the Samford for You" (SAASY) program included a 6-week acting class and four public performances. The acting class, led by two professional, met for a 2-hour class weekly for six weeks.	Mixed methods study with a single-group pre–post study design and interviews	12 older adults with chronic conditions from a low-income senior living community	The program led to significant improvements in psychological well-being and physical health-related quality of life among older adults with chronic conditions. The study found no significant improvement in the mental health component of the 36-Item Short-Form Health Survey after participation. Qualitative analysis highlighted enhanced self-worth, self-advocacy, and overcoming self-imposed limitations.
**Drama program**	(Holm et al., 2005; Lepp et al., 2003)	Drama program for dementia care	A drama program incorporating dance, rhythm, song, storytelling, and conversations. Sessions were led by trained drama educators and storytellers.	Qualitative Study	12 patients (10 women) with moderate to severe dementia and their 7 female caregivers; weekly 1.5-hour sessions over 2 months. .	Patients displayed increased self-confidence, open emotional expression (joy and sorrow), and greater interest in their surroundings. Caregivers reported strengthened professional identity, enhanced understanding of their roles, and deeper connections with the patients.<br>The storytelling invited the patients to take part in associative conversations.

**Drama therapy**	(Harel & Keisari, 2023)	Tele-drama therapy: Remote drama therapy sessions via telephone	Drama therapy techniques adapted to phone settings, focusing on embodiment, projection, and role-play to support older adults during COVID-19 lockdowns.	Qualitative study	19 community-dwelling older adults (mean age: 72.8 years), Hebrew and Arabic speakers.	The creative methods enriched the therapeutic sessions and promoted the therapeutic process. In addition, the contribution of the drama therapy methods was maintained in the phone setting. Despite the limitations and the restricted creative space on the phone, the participants devised new creative methods of embodiment, projection, and role, which enabled several significant therapeutic processes to occur.
**Drama therapy**	(Johnson, 1986)	Drama therapy	A program using developmental transformation, an improvisational approach to drama therapy in which two or more participants engage in a continuous improvisational, pretend play	A case study	Residents in a nursing home setting	This case study aimed to showcase various techniques within the Developmental Transformations (DvT) approach as applied in a nursing home setting. The therapy facilitated meaningful interpersonal relationships, reducing isolation and fostering playfulness and social cohesion. It supported emotional expression and integration, helping participants address complex feelings related to end of life, dependency, family dynamics, and life transitions.
**Drama therapy**	(Jaaniste et al., 2015)	drama therapy for dementia care	Drama therapy sessions involving storytelling, role-playing, and improvisation for 16 sessions over four months, compared to a	Controlled pre-post study design	13 participants with mild to moderate dementia	Average QoL scores improved for the drama therapy group and decreased for the movie group, though differences were not statistically significant. Participants expressed improved

			movie-watching control group. Activities focused on creativity, engagement, and personal expression.		(aged 61–88). The drama therapy group (N = 4) was compared with a group of participants (N = 9) who watched movies over a four-month period.	creativity, self-awareness, and engagement
**Drama therapy**	(Keisari & Palgi, 2017)	Life-Review and Drama Therapy Integration	A 12-session therapeutic intervention combining life-review and drama therapy, focusing on significant life events ("life-crossroads") and dramatizing them on stage to explore personal identity and well-being.	Controlled pre-post study design	55 older adults (ages 62–93) with normal cognitive functioning (MMSE > 24), divided into an intervention group (*n*=27) and a control group (*n*=28).	Significant improvements in meaning in life, self-acceptance, relationships with others, and successful aging. Depressive symptoms decreased among the intervention group.
**Drama therapy**	(Dassa & Harel, 2019)	Integration of music and drama therapy	A 10-session program combining music and drama therapy with performing arts students, culminating in an autobiographical	Qualitative study	12 people with dementia (ages 71–89, MMSE 6–19) and 12	Increased emotional expression, autonomy, dignity, and self-worth in participants with dementia. Strengthened intergenerational relationships and mutual learning.

			therapeutic theatre performance. Sessions included singing, acting, and collaborative improvisation to enhance self-expression and connection.		performing arts students (ages 23–27), working in pairs in a nursing home.	
**Drama therapy**	(Mechaeil et al., 2009)	Drama Therapy for Dementia care	A 12-week program comparing a Dramatherapy group with an activity control group. Dramatherapy included storytelling, role-play, and group exercises focusing on emotional expression, social interaction, and engagement.	Controlled pre-post study design	8 participants (ages 85–89); 4 in the Dramatherapy group and 4 in the control group, living in a care home.	Dramatherapy participants showed significant improvements in attentiveness and engagement compared to the control group. Effects were cumulative, suggesting long-term benefits from Dramatherapy interventions.
**Drama therapy**	(Leshem & Harel, 2023)	Drama Therapy and Life-Stories Group	A 10-month drama therapy program for older adults at a daycare center in Israel. Weekly 2-hour sessions included movement, music, and creative storytelling, with a focus on exploring and transforming life stories through dramatic resonances.	A case study	One group with twelve participants (aged 70–90) with mixed cognitive and physical abilities, ranging from independent individuals to those requiring assistance.	The therapists described how participants experienced increased self-expression, emotional processing, and interpersonal connection. Dramatic tools helped participants gain insights, accept past experiences, and improve their self-image.

**Drama therapy**	Harel, 2024	Therapeutic puppetry drama therapy for people with dementia	A 6-month drama therapy intervention using puppetry to support emotional expression, memory, and self-identity in older adults with dementia. Participants created puppets representing personal stories and used them in performances and storytelling.	A Case Study	3 participants (ages 67–92) with mild to moderate dementia, paired with 3 drama therapy MA students in an Israeli adult daycare center.	Puppetry encouraged creativity, supported memory recall, enhanced self-expression, and fostered emotional transformation, promoting well-being and interpersonal connection.
**Improvisational story telling**	(Swinnen & De Medeiros, 2018)	Participatory arts programs for people with dementia	Analysis of two programs: TimeSlips (creative storytelling) and the Alzheimer's Poetry Project (collaborative poetry creation). Both programs engage people with dementia in playful and imaginative activities to foster creativity and self-expression.	Humanities-Based Inquiry	Participants living with dementia in community settings or care facilities	Playful engagement led to increased joy, enhanced social connections, and improved well-being. The authors emphasized the importance of incorporating play into dementia care practices to enrich participants' lives meaningfully.
**Short playful interactions**	(Abu Elheja et al., 2021)	The mirror game	The mirror game is a non-verbal, dyadic interactive activity designed to promote coordination and synchronization between participants. It involves three stages: one participant leads while the other follows, roles are then switched, and finally,	A single-group pre–post study design	63 older adults (M = 78.93, SD = 9.99, range 65–101) completed emotional and social loneliness scales and	The results indicated that a short-term mirror-game of 15 minutes with older adults led to a reduction in sense of loneliness and changes in levels of saliva oxytocin. More specifically, the results showed that changes in oxytocin predicted a reduction in emotional loneliness in cases where individuals

			both participants synchronize movements without a designated leader. The game fosters a sense of connection and closeness.		provided saliva samples pre- and post-game to measure oxytocin (OT) levels.	experienced high levels of closeness and synchrony during the mirror game
**Short playful interactions**	(Keisari, Feniger-Schaal, et al., 2022)	The mirror game	Participants engaged in a 9-minute dyadic synchronized movement exercise, where they imitated each other's movements in three rounds: leader-follower, role-reversal, and mutual improvisation without a leader. This emphasized social and cognitive synchrony.	RCT in a within-subjects experimental design, where each participant engaged in two conditions: the mirror game and a control session (exercise class).	34 older adults (aged 71–98), 26 women, all cognitively healthy (MMSE > 24)	Mirror games significantly improved scores in attention, positive mood, and perceived social connection compared to the control. Reaction times in a spoken word-in-noise task were faster post-intervention.
**Short playful interactions**	(Golland et al., 2024 ; Keisari et al., 2024)	A short series of exercises based on improvisational theater	A short series of dyadic interactions lasting 15 minutes, based on improvisational theater (including activities such as the association game, the mirror game, and movement storytelling).	RCT, in a within-subjects experimental design, where each participant engaged in two conditions: a playful interaction session and a control session. The playful interaction included activities based on improvisational theater, while the	67 community-dwelling older adults, aged 75 to 100 (M = 88, SD = 5.7), all with normal cognitive functioning (MMSE > 24).	Results indicate that improvised playful interaction significantly improved performance on forward and backward digit span tasks, as well as word fluency, compared to the control condition. Furthermore, this interaction enhanced perceived partner responsiveness and led to increases in positive mood and subjective arousal relative to the control condition.

				control condition consisted of introductory dialogue, an exercise class, and verbal expression tasks. To examine the effect of delivery mode, half of the participants completed the sessions via Zoom (remote), and the other half participated face-to-face (F2F)		
**Short playful interactions**	(Benjamini et al., under review)	Short playful interactions	Short playful dyadic interactions lasting 15 minutes, based on improvisational theater (core activity was the movement storytelling).	RCT in a within-subjects experimental design, where each participant engaged in two conditions: the playful interaction and a control session (personal conversation and an exercise class).	34 older adults (aged 74–91, mean age=85), 24 women, all cognitively healthy (MMSE > 24)	A significant interaction (Time X Type of Activity) was found, validating the playful interaction's positive effect on increasing Digit Span score. A significant increase was also found in the social measures (closeness and affiliation) following the playful interaction but not following the control condition. There was no significant interaction for the Stroop or for positive and negative affect.
**Red Hat Society (RHS)**	(Chang & Yarnal, 2018)	Playfulness-Based Social Activities in the Red Hat Society	Focused on leisure-based social activities in the Red Hat Society (RHS), where women aged 50+ engaged in playful events such as dressing up in vibrant costumes, role-playing,	Longitudinal Study	167 women aged 51–80+, with varying levels of education, marital status, and	Increased playfulness over time significantly predicted resilience growth. Positive emotions and social interactions in RHS activities supported personal resource building and coping mechanisms.

			and participating in humorous and imaginative group games. These activities encouraged fun, laughter, and creative expression in a non-judgmental social environment.		health, participating in RHS activities for over 12 months.	
**Red Hat Society (RHS)**	(Yarnal et al., 2008)	Playfulness-Based Social Activities in the Red Hat Society	Focused on leisure-based social activities in the Red Hat Society (RHS), where women aged 50+ engaged in playful events such as dressing up in vibrant costumes, role-playing, and participating in humorous and imaginative group games. These activities encouraged fun, laughter, and creative expression in a non-judgmental social environment.	Mixed-Methods Study	1,693 women aged 50+ participating in RHS activities; data collected via online surveys and qualitative responses.	Play was defined as fun, laughter, and silliness. Participants reported benefits including increased joy, strengthened friendships, improved emotional well-being, and greater life satisfaction. Membership fostered a sense of identity and empowerment.

References

Abu Elheja, R., Palgi, Y., Feldman, R., Zagoory-Sharon, O., Shamay-Tsoory, S., & Keisari, S. (2021). The role of oxytocin in regulating loneliness in old age. *Psychoneuroendocrinology*.

Bassis, D., Rybko, J., & Maor, R. (2022). It's never too late to improvise: The impact of theatre improvisation on elderly population. *Experimental Aging Research*, 1–17. https://doi.org/10.1080/0361073X.2022.2059208

Benjamini, H., Keisari, S., Golland, Y., & Ben-David, B. M. (under review). The effect of online playful interactions on social, emotional and cognitive functioning among older adults. *Humanities and Social Sciences Communications*.

Chang, P.-J., & Yarnal, C. (2018). The effect of social support on resilience growth among women in the Red Hat Society. *The Journal of Positive Psychology*, *13*(1), 92–99. https://doi.org/10.1080/17439760.2017.1374442

Chung, K. S. Y., Lee, E. S. L., Tan, J. Q., Teo, D. J. H., Lee, C. B. L., Ee, S. R., Sim, S. K. Y., & Chee, C. S. (2018). Effects of Playback Theatre on cognitive function and quality of life in older adults in Singapore: A preliminary study. *Australasian Journal on Ageing*. https://doi.org/10.1111/ajag.12498

Dassa, A., & Harel, D. (2019). People with dementia as 'spect-actors' in a musical theatre group with performing arts students from the community. *The Arts in Psychotherapy*, 101592–101592. https://doi.org/10.1016/j.aip.2019.101592

Dunford, C. M., Yoshizaki-Gibbons, H. M., & Morhardt, D. (2017). The Memory Ensemble: Improvising connections among performance, disability, and ageing. *Research in Drama Education: The Journal of Applied Theatre and Performance*, *22*(3), 420–426. https://doi.org/10.1080/13569783.2017.1326806

Elkarif, T., Orkibi, H., & Keisari, S. (2024). Tele-drama therapy for community dwelling older adults with constricted life-space mobility: A randomized controlled trial. *The Journal of Positive Psychology*. https://doi.org/10.1080/17439760.2024.2427581

Golland, Y., Ben-David, B., & Keisari, S. (2024, May). *Brains on Playfulness: Playful interactions enhance cognition in older ages*. [Paper presented]. 7th Bi-annual Conference of the European Society for Cognitive and Affective Neuroscience (ESCAN), Ghent, Belgium.

Hafford-Letchfield, T. (2013). Funny things happen at the Grange: Introducing comedy activities in day services to older people with dementia – innovative practice. *Dementia*, *12*(6), 840–852. https://doi.org/10.1177/1471301212454357

Harel, D., & Keisari, S. (2023). Tele-drama therapy with community-dwelling older adults: A field training project. *Clinical Gerontologist*, *46*(3), 400–412. https://doi.org/10.1080/07317115.2023.2178353

Holm, A., Lepp, M., & Ringsberg, K. C. (2005). Dementia: Involving patients in storytelling – a caring intervention. A pilot study. *Journal of Clinical Nursing*, *14*(2), 256–263. https://doi.org/10.1111/j.1365-2702.2004.01042.x

Jaaniste, J., Linnell, S., Ollerton, R. L., & Slewa-Younan, S. (2015). Drama therapy with older people with dementia-Does it improve quality of life? *Arts in Psychotherapy*, *43*, 40–48. https://doi.org/10.1016/j.aip.2014.12.010

Johnson, D. R. (1986). The developmental method in drama therapy: Group treatment with the elderly. *The Arts in Psychotherapy*, *13*(1), 17–33. https://doi.org/10.1016/0197-4556(86)90005-5

Keisari, S., Feniger-Schaal, R., Palgi, Y., Golland, Y., Gesser-Edelsburg, A., & Ben-David, B. (2022). Synchrony in old age: Playing the mirror game improves cognitive performance. *Clinical Gerontologists*, *45*(2), 312–326.

Keisari, S., Gesser-Edelsburg, A., Yaniv, D., & Palgi, Y. (2020). Playback theatre in adult day centers: A creative group intervention for community-dwelling older adults. *PLOS ONE*, *15*(10), e0239812–e0239812. https://doi.org/10.1371/journal.pone.0239812

Keisari, S., Golland, Y., & Ben-Ari, B. (2024, May). *Improvised playful interactions with older adults: Effects on cognitive and social performance*. American Psychological Association (APA) Convention, Seattle, WA.

Keisari, S., & Palgi, Y. (2017). Life-crossroads on stage: Integrating life review and drama therapy for older adults. *Aging and Mental Health*, *21*(10). https://doi.org/10.1080/13607863.2016.1199012

Keisari, S., Palgi, Y., Yaniv, D., & Gesser-Edelsburg, A. (2022). Participation in life-review playback theater enhances mental health of community-dwelling older adults: A randomized controlled trial. *Psychology of Aesthetics, Creativity, and the Arts*. https://doi.org/10.1037/aca0000354

Keisari, S., Yaniv, D., Gesser-edelsburg, A., Palgi, Y., Neimeyer, R. A., Keisari, S., Yaniv, D., Gesser-edelsburg, A., Palgi, Y., & Neimeyer, R. A. (2023). *Psychotherapy Meaning Reconstruction 70 Years Later: Processing Older Adults ' Unfinished Business in a Drama Therapy Group EVIDENCE-BASED CASE STUDY Meaning Reconstruction 70 Years Later: Processing Older Adults ' Un fi nished Business in a Drama Thera*.

Keisari, S., Yaniv, D., Palgi, Y., & Gesser-Edelsburg, A. (2018). Conducting playback theatre with older adults—A therapist's perspective. *Arts in Psychotherapy*, *60*, 72–81. https://doi.org/10.1016/j.aip.2018.07.002

Lepp, M., Ringsberg, K. C., Holm, A., & Sellersjö, G. (2003). Dementia – involving patients and their caregivers in a drama programme: The caregivers' experiences. *Journal of Clinical Nursing*, *12*(6), 873–881. https://doi.org/10.1046/j.1365-2702.2003.00801.x

Leshem, E., & Harel, D. (2023). A patchwork quilt: Drama therapy and life-stories group with older adults. *GeroPsych*. https://doi.org/10.1024/1662-9647/a000325

Lindquist, L. A., Liggett, A., Muhammad, R., Seltzer, A., Kim, K.-Y. A., Barish, B., Wagner, A., & Ramirez-Zohfeld, V. (2021). Effects of improv training on older adults in a long term care facility. *Https://Doi-Org.Ezproxy.Haifa.Ac.Il/10.1177/23337214211016111*, *7*. https://doi.org/10.1177/23337214211016111

Mechaeil, R., Graybow, A., & Cobham, P. (2009). Towards a Purposeful Ending: A Study Exploring the Impact of Dramatherapy on the Quality of Life of People Suffering from Dementia. *Dramatherapy*, *31*(2), 27–34. https://doi.org/10.1080/02630672.2009.9689775

Morse, L. A., Xiong, L., Ramirez-Zohfeld, V., Anne, S., Barish, B., & Lindquist, L. A. (2018). Humor doesn't retire: Improvisation as a health-promoting intervention for older adults. *Archives of Gerontology and Geriatrics*, *75*, 1–5. https://doi.org/10.1016/J.ARCHGER.2017.10.013

Noice, H., & Noice, T. (2013). Extending the Reach of an evidence-based theatrical intervention. *Experimental Aging Research*, *39*(4), 398–418. https://doi.org/10.1080/0361073X.2013.808116

Noice, T., & Noice, H. (2021). A theatrical evidence-based cognitive intervention for older adults. *Drama Therapy Review*, *7*(1), 9–22. https://doi.org/10.1386/dtr_00058_1

Noice, T., Noice, H., & Kramer, A. F. (2015). Theatre arts for improving cognitive and affective health. *Activities, Adaptation and Aging*, *39*(1), 19–31. https://doi.org/10.1080/01924788.2015.994440

Stevens, J. (2012). Stand up for dementia: Performance, improvisation and stand up comedy as therapy for people with dementia; a qualitative study. *Dementia*. https://doi.org/10.1177/1471301211418160

Sutherland, L., Dunkle, R. E., & Pace, G. T. (2024). Enhancing social connections through an acting and improvisation course for older Americans in low-income housing. *Arts & Health*, *16*(1), 48–63. https://doi.org/10.1080/17533015.2023.2211085

Swinnen, A., & De Medeiros, K. (2018). "Play" and people living with dementia: A humanities-based inquiry of TimeSlips and the Alzheimer's poetry project. *Gerontologist*, *58*(2), 261–269. https://doi.org/10.1093/geront/gnw196

Woslov, C., Keisari, S., & Harel, D. (2024). 'I see them speak, I can hear their movements': Playfulness and personal growth in playback theatre groups for older women. *Dramatherapy*, *44*, 71–89.

Yamamoto, R. H. (2020). Improv as creative aging: The perceived influences of theatrical improvisation on older adults. *Activities, Adaptation & Aging*, *45*, 217–233. https://doi.org/10.1080/01924788.2020.1763075

Yarnal, C. M., Chick, G., & Kerstetter, D. L. (2008). "I did not have time to play growing up... so this is my play time. It's the best thing i have ever done for myself": What is play to older women? *Leisure Sciences*, *30*(3), 235–252. https://doi.org/10.1080/01490400802017456

Yuen, H. K., Mueller, K., Mayor, E., & Azuero, A. (2011). Impact of participation in a theatre programme on quality of life among older adults with chronic conditions: A pilot study. *Occupational Therapy International*, *18*(4), 201–208. https://doi.org/10.1002/oti.327

Zeisel, J., Skrajner, M. J., Zeisel, E. B., Wilson, M. N., & Gage, C. (2018). Scripted-IMPROV: Interactive improvisational drama with persons with dementia—Effects on engagement, affect, depression, and quality of life. *American Journal of Alzheimer's Disease and Other Dementias*. https://doi.org/10.1177/1533317518755994
